# Clinical Named Entity Recognition from Chinese Electronic Medical Records Based on Deep Learning Pretraining

**DOI:** 10.1155/2020/8829219

**Published:** 2020-11-24

**Authors:** Lejun Gong, Zhifei Zhang, Shiqi Chen

**Affiliations:** ^1^Jiangsu Key Lab of Big Data Security & Intelligent Processing, School of Computer Science, Nanjing University of Posts and Telecommunications, Nanijing 210023, China; ^2^Zhejiang Engineering Research Center of Intelligent Medicine, Wenzhou 325035, China

## Abstract

**Background:**

Clinical named entity recognition is the basic task of mining electronic medical records text, which are with some challenges containing the language features of Chinese electronic medical records text with many compound entities, serious missing sentence components, and unclear entity boundary. Moreover, the corpus of Chinese electronic medical records is difficult to obtain.

**Methods:**

Aiming at these characteristics of Chinese electronic medical records, this study proposed a Chinese clinical entity recognition model based on deep learning pretraining. The model used word embedding from domain corpus and fine-tuning of entity recognition model pretrained by relevant corpus. Then BiLSTM and Transformer are, respectively, used as feature extractors to identify four types of clinical entities including diseases, symptoms, drugs, and operations from the text of Chinese electronic medical records.

**Results:**

75.06% Macro-*P*, 76.40% Macro-*R,* and 75.72% Macro-*F*1 aiming at test dataset could be achieved. These experiments show that the Chinese clinical entity recognition model based on deep learning pretraining can effectively improve the recognition effect.

**Conclusions:**

These experiments show that the proposed Chinese clinical entity recognition model based on deep learning pretraining can effectively improve the recognition performance.

## 1. Background

In recent years, medical informatization has produced a large number of electronic medical records. The electronic medical record not only completely preserves the detailed information of the patients' diagnosis and treatment process but also has the advantages of regular writing format, convenient retrieval, and storage, and it can better help telemedicine further. In addition, the rapid development of a large number of online consultation websites and cases discussion forums will also produce a large number of disease question and answer information. These medical texts are in the same form as electronic medical records. These data make up a very large amount of medical data resources.

Electronic medical records (EMR) record various symptoms and examination measures taken by patients from before admission to hospitalization and that medical personnel provides based on examination results such as disease diagnosis and treatment methods as medical resources constructed by professionals. As the core data of medical information system, how to make use of the large amount of potential medical information contained in electronic medical records has become one of the hot research directions. But electronic medical records are not fully structured data. Semistructured or unstructured free text data make up the majority. In order to convert these unstructured data into structured form that can be recognized by computer, it is necessary to use natural language processing technology to conduct text mining. As the basic task of text mining information extraction, the types of clinical entities to be recognized mainly include disease, symptoms, operations taken by medical personnel (including inspection operations and treatment operations), and drugs. Although the research on Chinese named entity recognition has been going on for some time, most of the researches focus on the open field. However, some studies have shown that the density of entity distribution in Chinese EMRs is much higher than that in open field texts. The proportion of entity characters in the corpus of Chinese EMRs is nearly twice that of the general Chinese corpus, which indicates that Chinese EMRs are a kind of knowledge-intensive text [1], and the data has considerable research value. But this density also creates more obstacles to the study of clinical named entity recognition from EMRs in Chinese. Since it is the entity recognition of Chinese electronic medical record, this paper keeps the entity in Chinese medical record as Chinese character format. This task has just started. In addition, it still has the following difficulties [2].

1. Clinical entities have various types and a large number, and there are new entities with unregistered words, such as unregistered disease, drug, and inspection, which make it difficult to build a comprehensive clinical dictionary and obtain disease dictionary, drug dictionary, or inspection dictionary.
2. Clinical entities are divided into simple entities and complex entities with relatively complex structure. The length of medical record entities in EMR is variable, and a large number of clinical entities are longer than common entities. There are a lot of nesting, alias, and acronym in clinical entities.
3. In different parts of EMR, the extension of clinical entity is different, and there is fuzzy classification in category labeling. The boundary between different named entities is not clear, and the names of clinical manifestations often appear in the names of diseases. There are a lot of mutual inclusion and crossover phenomenon. For example, “上呼吸道感染” is generally considered to be the disease, but, in some cases, it also appears as a symptom.

The recognition of named entity in EMR has been studied in foreign countries. Because EMR involves more professional knowledge, the cost of corpus construction is higher. The informatics for Integrating Biology and the Bedside (I2B2) organized the multiple records related tasks and issued the relevant corpus and a number of shared tasks since 2006 [3]. The task of concept recognition and relation extraction evaluation of I2B2 2010 is the first to systematically classify English electronic medical record name entities. This classification refers to the semantic type defined by UMLS, which divides EMR entities into three categories, namely, medical problems (including diseases and symptoms), treatment, and examination.

Named entity recognition, as the key task of text data mining, has been the research foundation and hotspot of natural language processing. Named entity recognition in the general field mainly includes names, places, organizations, time expressions, and numerical expressions. In the field of biomedicine, most of the current research focuses on the identification of genes, proteins, cells, and other entities in the English medical literature. Although the specific objects identified are different, a large number of entity identification methods in the general field can still be applied to the biomedical field. These include early approaches based on the combination of dictionaries and rules and approaches based on machine learning.

The method based on the combination of dictionary and rule will match the dictionary and text firstly. Then it combines with the formulated rules for postprocessing and normalization. Its performance depends on the size of the dictionary and the quality of the rules [4]. Due to the variety of clinical entities and its strong professionalism, the construction of dictionaries and the formulation of rules need amount manpower, which is not only time-consuming, but also not portabl e. Therefore, dictionaries and rules are often used as auxiliary means in the task of named entity recognition.

In recent years, machine learning has been applied to named entity recognition [5, 6], such as maximum entropy (ME), conditional random field (CRF), support vector machine (SVM), and structural support vector machine (SSVM). Multiple deep learning methods are also applied to entity recognition tasks. For example, Recurrent Neural Networks (RNN) and Long Short-Term Memory Networks (LSTM). In addition, models combining deep learning with traditional machine learning have also been widely used.

Named entity recognition is often regarded as a sequential annotation task. Traditional machine learning methods, such as CRF [7], can achieve good performance in sequence annotation tasks but rely heavily on manually selected characteristics. By contrast, deep learning could automatically learn features, but a large amount of training data is needed to achieve excellent recognition effect [8, 9].

Related work of Chinese EMR is developing rapidly [10, 11]. Compared with English corpus, Chinese text has fuzzy word boundary and no obvious segmentation mark, so it is difficult to study the entity recognition [12]. The selection of features in traditional machine learning will directly affect the effect of entity recognition, so most studies focus on the construction and selection of different features. Lei et al. [13] compared the combination of CRF, SSVM, SVM, and ME with a variety of characteristics and recognized medical problems, examination, treatment, and drugs in the admission records and discharge records. Wang et al. [14] used the character position information and short clauses to reach the *F*1 value of 95.12% in the self-labeled Chinese medicine text corpus. Literature [15] studies the influence of multifeature combination such as linguistic symbol feature, part of speech feature, keyword feature, and dictionary feature on CRF sequence annotation.

There are also relevant studies on Chinese clinical entity recognition using the deep learning method [16–18], whose model is basically the sequence model RNN and its variants.

It is worth mentioning that Yang et al. [19] combined the characteristics of Chinese electronic language structure and, with the help of the guidance of professional medical personnel, combined EMR label specification in English and made a detailed EMR in Chinese named entity and entity relationship labeling regulations and completed the natural language processing research in the field of EMR in Chinese basic work. In addition, there are some identification methods that combine deep learning with supervised learning [20, 21]. However, as far as we know, BiLSTM and Transformer [22] combined methods have not been applied to clinical Chinese-named entity recognition.

In view of the above problems, this study proposes a named entity recognition method for Chinese EMR based on pretraining. The method is based on word embedding pretraining and fine-tuning of entity recognition model pretrained by relevant corpus. BiLSTM and Transformer are, respectively, used as feature extractors to effectively recognize clinical entities in Chinese EMR.

## 2. Methods

The problem of Chinese clinical entity recognition can be transformed into sequence labeling. Sequence annotation problem is to determine the output tag sequence B=*b*_1_,…, *b*_*n*_(*b*_*i*_ ∈ *L*, 1 ≤ *i* ≤ *n*) for input sequence *A*=*a*_1_, *a*_2_, *a*_3_, ..., *a*_*n*_ and tag set L. Its essence is to classify each element in the input sequence according to its context.

**Figure 1:**
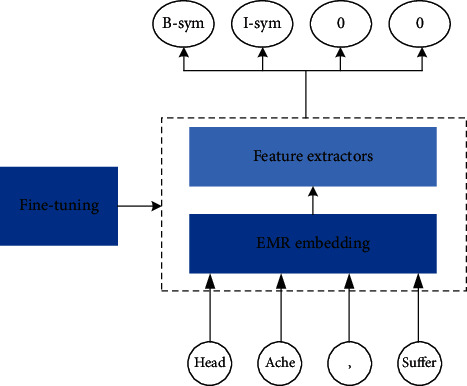
Pipeline of deep learning pretraining.

There were two specific practices for implementing the deep learning pretraining mode: firstly, the input is initialized by the same field corpus pretraining EMR embedding and, secondly, the entity recognition model pretrained by relevant corpus is fine-tuning. We studied the effect of this model on the recognition of clinical entities as shown in Figure 1.

### 2.1. Datasets

Because of the protection policy of patient privacy in China, it is difficult to obtain electronic medical records in hospitals. Therefore, we got 1,064 respiratory records and 30,262 unrestricted records were crawled from the website (https://www.iiyi.com). 200 of the 1,064 respiratory department EMRs were manually annotated according to the annotation specification shown in Table 1 based on [19] and the semantic types of English I2B2 and UMLS, indicating four medical entities of disease, symptom, drug, and operation.  Table 2 shows the distribution of training set and test set.

Skip-gram model of Word2vec was used to adopt the EMR word embedding from 30,262 sets of unmarked electronic medical records (115 MB), called the first dataset. In addition, in order to study the impact of word embedding language on downstream task, we also use the universal word embedding with 268G news corpus, called the second dataset.

For the sequential annotation task of entity recognition, the tag is composed of two parts: the entity category and the location in the entity. In this study, BIO representation is used to represent the entity category and the position of the entity and then character as the minimum annotation unit. In the BIO representation, B is at the beginning of the entity, I is inside the entity, and O is not an entity. Therefore, the labeled corpus contains 4 types of entities and 9 types of labels.

### 2.2. Pretraining

There is a pretraining with fine-tuning mode in addition to the character embedding of Chinese EMR. It is difficult to annotate the corpus of Chinese EMRs. In order to make full use of the resources of previous studies, it is used to fine-tune our recognition tasks based on a clinical entity recognition model (https://github.com/baiyyang/medical-entity-recognition) trained by medical data of CCKS2017 tasks. This model uses BiLSTM as feature extractor followed by CRF for sequence annotation. For the convenience of description, this paper calls it as BioModel.

Although the labeling target of BioModel is different from our task. However, Chinese EMR texts all have the same linguistic features, and the model's ability to learn this language feature can be well transferred to our task.

### 2.3. Bidirectional Long Short-Term Memory-Conditional Random Fields

In recent years, a variety of deep learning methods have been widely applied in named entity recognition tasks, usually using RNN model and its variants. RNN is theoretically capable of capturing remote context relationships, but, in practice, RNN cells often fail due to gradient disappearance or gradient explosion. Therefore, LSTM is usually used in practical applications.

**Figure 2:**
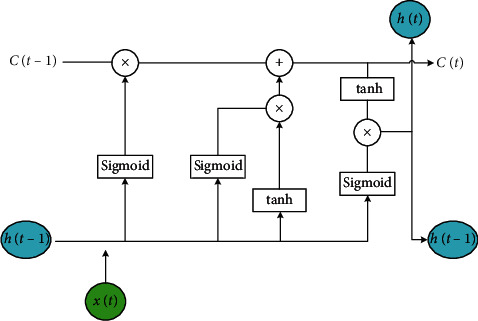
LSTM cell.

LSTM uses a separate update gate Γ_u_ and a forget gate Γ_f_, as well as an output gate Γ_o_. The update gate selectively updates the state of the current moment, while the forget gate selectively forgets the state of the previous moment. And then the output gate controls the proportion of the output of the current state.  Figure 2 depicts the internal structure of an LSTM cell [6]. The realization of LSTM is as follows:

(1) $$\begin{array}{c} \tilde{C}\left(t\right)=\tanh\left(W_{c}\left[h\left(t-1\right),x\left(t\right)\right]+b_{c}\right),\\ \Gamma_{\text{u}}=\text{sigmoid}\left(W_{u}\left[h\left(t-1\right),x\left(t\right)\right]+b_{u}\right),\\ \Gamma_{\text{f}}=\text{sigmoid}\left(W_{f}\left[h\left(t-1\right),x\left(t\right)\right]+b_{f}\right),\\ \Gamma_{\text{o}}=\text{sigmoid}\left(W_{o}\left[h\left(t-1\right),x\left(t\right)\right]+b_{o}\right),\\ C\left(t\right)=\Gamma_{\text{u}}\tilde{C}\left(t\right)+\Gamma_{\text{f}}C\left(t-1\right),\\ h\left(t\right)=\Gamma_{\text{u}}*\;\tanh\left(C\left(t\right)\right). \end{array}$$

In the named entity recognition task, simultaneous access to the context of the current moment can help predict the current moment. However, LSTM's hidden state *h*(*t*) accepts only past information. Therefore, we use a bidirectional LSTM model to give the context of each state, using two independent hidden states from left to right and from right to left, while capturing past and future information.

**Figure 3:**
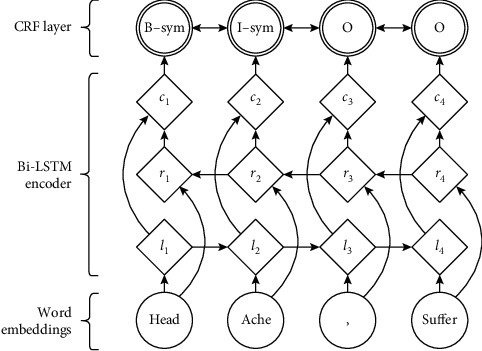
BiLSTM-CRF.

BiLSTM converts the input sequence through the embedding layer into a vector sequence input into two LSTM networks and then contact the forward and reverse two hidden layer outputs into the Softmax layer for classification. However, LSTM can only learn the context relation of features but cannot directly learn the context relation of tags. Without the constraint of state transition, the model is likely to output a completely wrong tag sequence. Therefore, it is considered to replace Softmax layer with CRF layer. CRF is still responsible for sequence annotation, and BiLSTM is responsible for automatic feature selection.  Figure 3 describes the BiLSTM-CRF model used in the clinical entity recognition.

### 2.4. Transformer-Conditional Random Fields

Although the structure of gate mechanism such as LSTM alleviates the problem of long-term dependence to some extent, LSTM is still unable to do anything for the special long-term dependence phenomenon. The calculation of LSTM is limited to sequence; that is, it can only be calculated in sequence from left to right or from right to left, and the loss of information in the process of sequential calculation is inevitable.

Transformer solves this problem by using the attention to reduce the distance between any two positions in the sequence to a constant. Therefore, Transformer, as a feature extractor, has a stronger learning ability than LSTM and has been widely used in the past two years.

**Figure 4:**
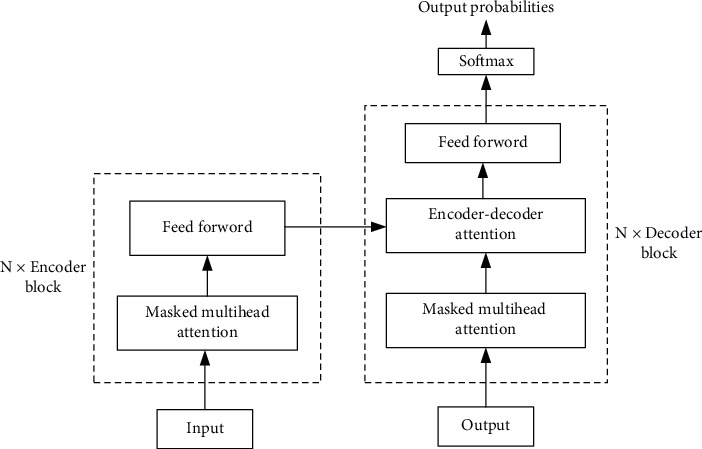
Transformer.

As shown in Figure 4, Transformer is stacked with encoder and decoder, and, like all generation models, the output of the encoder is the input of the decoder [12]. All encoder blocks are structurally identical, but they do not share parameters. Each encoder block can be decomposed into two sublayers, composed of self-attention and Feed Forward Neural Network. After the data is passed through the self-attention module, the weighted feature vector *Z* is obtained and then sent to the next module of encoder block, namely, Feed Forward Neural Network, to obtain the output FFN (*Z*) of an encoder block.

(2) $$\begin{array}{c} Z=\text{Attention}\left(Q,K,V\right)=\text{soft}\max\left(\frac{QK^{T}}{\sqrt{d_{k}}}\right)V,\\ \text{FFN}\left(Z\right)=\max\left(0,ZW_{1}+b_{1}\right)W_{2}+b_{2}. \end{array}$$

Among them, *Q*, *K,* and *V* are assumed to be composed of a series of <*Q*, *K*, *V*> data pairs. For any constituent element *Q*, the weight coefficient of each *K* corresponding to *V* can be obtained by calculating the similarity between the current element *Q* and other elements *K*, and then the weighted sum of *V* can be carried out to obtain the final attention value.

Decoder block has one more encoder-decoder attention than encoder. The two types of attention of decoder are used to calculate the weight of input and output, respectively. Self-attention is used to calculate the relationship between current output and preorder output. Encoder-decoder attention calculates the relationship between the current output and the encoder input eigenvector. In encoder-decoder attention, *Q* comes from the last output of decoder, and *K* and *V* come from the output of encoder.

Multihead self-attention represents the different ways of fusion of the target word and the semantic vector of other words in the text under various semantic scenes. Note that there are multiple sets of *Q*/*K*/*V* weight matrices in the mechanism, each of which is randomly initialized, and after training, each set is used to embed the input word or the vector from the previous encoder/decoder into a different representation subspace.

## 3. Results and Discussion

In order to comprehensively consider the performance of the model on the whole dataset, macroaverage is adopted in this paper. Macroaverage refers to the arithmetic average of each kind of performance indicator, which can be divided into macroprecision (Macro-*p*), macrorecall (Macro-*r*), and Macro-*F*1 (Macro-*F*1).

(3) $$\begin{array}{c} \text{Marco}-P=\frac{\sum_{i=1}^{N_{c}}P_{\text{i}}}{N_{c}},\\ \text{Marco}-R=\frac{\sum_{i=1}^{N_{c}}R_{\text{i}}}{N_{c}},\\ \text{Marco}-F1=\frac{2\times \text{Macro}-P\times \text{Macro}-R}{\text{Macro}-P+\text{Macro}-R}, \end{array}$$

*N*_c_ represents the total number of entity categories, *P*_i_ represents the precision of each category of entity, and *R*_i_ represents the recall of each category of entity.

In order to investigate the effect of BiLSTM-CRF embedding of different dimensions on the test set, we conducted this set of comparative experiments as shown in Table 3 using the first dataset as test dataset. From Table 3, if the dimension of word embedding is too small, the implied semantic information will be lost. If the dimension of word embedded is too large, it will bring noise. How to set the dimension of word embedding is related to the size and the language characteristics of the corpus.

In deep learning, the quality of word embedding has a great influence on the recognition results of deep neural network. In this study, the experiment effect of 150-dimension word embedding is the best. Therefore, two different word embeddings combined with BiLSTM-CRF and Transformer-CRF form the following four groups of experiments using the 150-dimension and the two types of dataset as shown in Table 4.

It can be seen from the results that EMR embedding has better common embedding than whatever model is used. Although the corpus size of EMR embedding is smaller than that of common embedding, the strong relevance of EMR embedding to downstream tasks makes the effect of EMR embedding significantly better than that of embedding with universal corpus.

In addition, by comparing the experimental results of BiLSTM-CRF and Transformer-CRF, it can be found that although the feature extraction ability of Transformer is theoretically better than that of BiLSTM, the complex model structure of Transformer requires a large amount of training data for learning. With the case of fewer training samples, Transformer does not perform as well as BiLSTM.

To study the effectiveness of pretraining with the fine-tuning related entity recognition model, EMR embedding was adopted to BioModel fine-tuning aiming at the first dataset, and the medical entity recognition model was obtained, called BioModel-fine. As the basic network structure of BioModel is BiLSTM-CRF, the experimental control group has also used the BiLSTM-CRF network model embedding EMR. The performances of comparisons between pretraining and not pretraining is shown in Table 5.

BioModel has achieved 79% Macro-*p*, 80% Macro-*r,* and 80% Macro-*F*1 on its original test set in CCKS2017. However, the recognition target of its original corpus is different from ours. Using the first dataset as test data, BioModel could obtain 72.48% Macro-*p*, 72.54% Macro-*r,* and 72.51% Macro-*F*1. BioModel-fine model obtains 75.06% Macro-*p*, 76.40% Macro-*r,* and 75.72% Macro-*F*1. The more details of BioModel-fine model are as shown in Tables 5 and 6.

**Figure 5:**
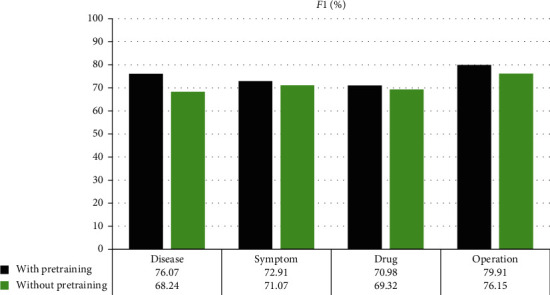
Two types of *F*1 comparison on entity.

BioModel-fine is the model structure based on BioModel for pretraining. Compared with the above results without pretraining with fine tuning, fine-tuning can significantly improve the experimental results by utilizing the implied information learned by BioModel from its own training data. Further, *F*1 is used to measure the performances between pretraining and nonpretraining as shown in Figure 5.

From the above, we can see that the most different difference between the pretraining and nonpretraining modes is the disease. It is mainly because the BioModel also recognized disease entity, and, for the disease entity, its word formation is similar, often ending with “病” and “症.” Further, the positions appearing in the EMR are relatively stable, and these characteristics can be well learned based on the context. Relatively, the smallest difference between the pretraining and nonpretraining modes is the drug. This is because drug entities are mostly unfamiliar, and the word formation is quite different from the free text of other parts of the medical record. It is difficult to learn, and the drugs appearing in EMR from different departments tend to be quite different, and the recognition of drug is very difficult, in essence.

In addition, in Chinese electronic medical records, there are a large number of long entities and even super long entities with characters longer than 10, such as “双侧腋下扪及黄豆大小淋巴结” and “右肺中叶大片密度增高阴影.” This paper also computes character length statistics for BioModel-fine entity recognition results with 4.63 average character. Though BiModel-fine model based on the deep neural network relies on the performance of adjacent words, the learned gate structure can retain more long-term effective information and has more advantages over the implied characteristics in long-term dependence. BioModel-fine has generally shown greater sensitivity to these medical entities with longer character lengths.  Table 7 lists the five examples of identifying long entities by BiModel-fine model.

## 4. Conclusions

In this study, a pretrained method for Chinese electronic medical record named entity recognition is proposed in view of the language features of Chinese EMR with many compound entities, serious missing sentence components, unclear entity boundary, and the difficulty in obtaining annotated corpus. Pretraining is divided into two steps. The first step is to adopt the same field of corpus pretraining word embedding and, respectively, use BiLSTM and Transformer as feature extractor to identify medical entities in Chinese electronic medical records. The second step is fine-tuning the named entity recognition model pretrained by other relevant corpus, so as to make full use of the existing annotated corpus and effectively improve the recognition effect of Chinese clinical entities when there are few annotated corpus. 75.06% Macro-*P*, 76.40% Macro-*R,* and 75.72% Macro-*F*1 could be achieved aiming at test dataset related to the Chinese electronic medical records. Experiment results show that the proposed Chinese clinical entity recognition model based on deep learning pretraining could effectively improve the recognition performance.

## Figures and Tables

**Table 1:** Labeling rules.

Entity types	Definition	Medical entities
Disease	The diagnosis made by doctors to patients or entities ending with “病” or “症” are collectively referred to as diseases.	肺内隔离症
Symptoms	Symptoms of discomfort, abnormalities, normal or abnormal examination results, or an unhealthy state of the patient, as well as the patient's self-reported history.	声音嘶哑、无结核病史
Drug	The specific drug name or class of drug given to the patient during treatment.	地塞米松、抗生素
Operation	This includes screening programs and treatments. A test item is given to a patient in order to discover, deny, confirm, and find out more about the disease. Treatment refers to the treatment procedures and interventions that are imposed on patients to solve the disease or relieve symptoms.	拍胸片、抗感染、胸腔穿刺术

**Table 2:** Distribution of entities among the training set and the test set.

Data	Disease	Symptoms	Drug	Operation	The total number of entities
Training set	701	2648	546	2138	6033
Test set	273	1043	208	918	2442

**Table 3:** Comparison results of different dimensions.

Different dimensions	Marco-*P* (%)	Marco-*R* (%)	Marco-*F*1 (%)
Random embedding	69.52	69.70	69.38
50 embeddings	53.42	54.31	53.74
**150 embeddings**	**72.48**	**72.54**	**72.51**
300 embeddings	55.36	61.03	57.88

**Table 4:** Comparisons of different recognition models and different word embedding.

Models	Dataset	Marco-*P* (%)	Marco-*R* (%)	Marco-*F*1 (%)
BiLSTM-CRF + embedding	Second	68.37	70.84	69.58
**BiLSTM-CRF** **+** **EMR embedding**	**First**	**72.48**	**72.54**	**72.51**
Transformer-CRF + embedding	Second	52.70	69.50	59.90
Transformer-CRF + EMR embedding	First	52.70	72.10	60.70

**Table 5:** Comparisons between pretraining and not pretraining.

Models	Marco-*P* (%)	Marco-*R* (%)	Marco-*F*1 (%)
BioModel	72.48	72.54	72.51
**BioModel-fine**	**75.06**	**76.40**	**75.72**

**Table 6:** Performances of BioModel-fine.

Types	*P* (%)	*R* (%)	*F*1 (%)
Disease	77.07	75.09	76.07
Drug	70.81	71.15	70.98
Operation	79.28	80.56	79.91
Symptom	71.74	74.12	72.91
**Average**	**75.06**	**76.40**	**75.72**

**Table 7:** Identifying five examples of long entities by BiModle-fine model.

No	Identified medical entities
1	双侧腋下扪及黄豆大小淋巴结 (symptom)
2	右肺中叶大片密度增高阴影 (symptom)
3	两肺纹理间可见边界不清的粟粒样微小淡结节影 (symptom)
4	急性心肌梗塞 (disease)
5	结核 PCR 扩增实验 (operation)

## Data Availability

The data used to support the findings of this study are available from the corresponding author upon request.
